# Research on Structure Optimization and Measurement Method of a Large-Range Deep Displacement 3D Measuring Sensor

**DOI:** 10.3390/s20061689

**Published:** 2020-03-18

**Authors:** Nanying Shentu, Sheng Wang, Qing Li, Renyuan Tong, Siguang An, Guohua Qiu

**Affiliations:** 1 College of Mechanical and Electrical Engineering, China Jiliang University, Hangzhou 310018, China; ws310545@163.com (S.W.); tongrenyuan@126.com (R.T.); annsg@163.com (S.A.); 2 College of Information Enginerrig, China Jiliang University, Hangzhou 310018, China; qghfr@163.com

**Keywords:** geological disaster, deep displacement monitoring, horizontal displacement, vertical displacement, mathematical modeling, contour

## Abstract

Deep displacement monitoring of rock and soil mass is the focus of current geological hazard research. In the previous works, we proposed a geophysical deep displacement characteristic information detection method by implanting magneto-electric sensing arrays in boreholes, and preliminarily designed the sensor prototype and algorithm of deep displacement three-dimensional (3D) measurement. On this basis, we optimized the structure of the sensing unit through 3D printing and other technologies, and improved the shape and material parameters of the permanent magnet after extensive experiments. Through in-depth analysis of the experimental data, based on the data query algorithm and the polynomial least square curve fitting theory, a new mathematical model for 3D measurement of deep displacement has been proposed. By virtue of it, the output values of mutual inductance voltage, Hall voltage and tilt measuring voltage measured by the sensing units can be converted into the variations of relative horizontal displacement, vertical displacement and axial tilt angle between any two adjacent sensing units in real time, and the measuring errors of horizontal and vertical displacement are tested to be 0–1.5 mm. The combination of structural optimization and measurement method upgrading extends the measurement range of the sensing unit from 0–30 mm to 0–50 mm. It shows that our revised deep displacement 3D measuring sensor can better meet the needs of high-precision monitoring at the initial stage of rock and soil deformation and large deformation monitoring at the rapid change and imminent-sliding stage.

## 1. Introduction

Geological disasters refer to rock and soil mass sliding accidents that damage ecological environment resources and seriously threaten human life and property under the influence of natural or human factors, including collapse, landslides, debris flows and so on [1,2,3,4,5,6]. According to the data, 70% of geological disasters are landslides and collapses. Landslide deformation is an integrated reflection of geological structure and internal and external influencing factors of a landslide mass. In view of the actual needs of disaster prevention and mitigation work, we should focus on the monitoring technology of rock and soil mass stability at present [7], which mainly includes surface displacement monitoring and deep displacement monitoring [8]. A large number of scholars’ research shows that the initial stage of the landslide is mainly characterized by deformation caused by shear force in the deep part of rock and soil mass, and with the gradual development of landslide, the final stage is characterized by cracks in the surface layer of rock and soil mass, followed by collapse [9]. Therefore, studying the deep displacement of rock and soil mass is more in line with the needs of disaster prevention and mitigation than the study of surface displacement. The deep displacement of rock and soil mass can reflect the dynamic stability of rock and soil mass more accurately and quickly, therefore it provides accurate, powerful data and theoretical support for early warning of landslides.

However, the deep displacement measuring device must be buried deep underground, requiring the instrument to be strong, anticorrosive, convenient for installation, operation, long-distance signal transmission and so on. At the same time, the deep displacement monitoring environment is harsh and complicated, and there are problems such as no light, water seepage, corrosion, geotechnical shearing and extrusion, which can easily cause damage to the buried instruments. Therefore, compared with surface displacement monitoring, the development of deep displacement monitoring technology is relatively slow, and the types of deep displacement monitoring instruments that can be practically applied are obviously less and have poorer performance. At present, the global monitoring of deep displacement of rock and soil mass mainly includes borehole inclinometer, time domain reflection technology, Brillouin Optical Time Domain Reflection (BOTDR) technology, borehole extensometer and sedimentation instrument.

Borehole inclinometer is the most mature and widely used deep displacement monitoring technology for rock and soil mass due to its simple structure, high reliability and ability to measure the deep horizontal displacement and velocity of rock and soil mass. However, there are still many shortcomings in the development of this technology [10,11]. Each measuring unit of the inclinometer has strong coupling and rigidity, which leads to the insensitivity to small displacement in the deep displacement measurement of the soft soil, that is, the sensitivity of the inclinometer is limited. At the same time, the strong coupling in the displacement of the rock and soil mass will make the inclinometer fracture and lose the monitoring function. Finally, the inclinometer cannot measure the vertical displacement of the rock and soil mass, which will result in the failure to monitor the three-dimensional displacement of the deep rock and soil, loss of important deep displacement information of geological mass and weakening the early warning function of the system.

The TDR technology has been developed due to its preferable precision, efficiency and automation degree in measurement process [12,13,14]. However, TDR technology still has its shortcomings. It is mostly used to measure soil shear force and hardly suitable for three-dimensional measurement of deep displacement of rock and soil [15,16]. TDR technology cannot measure the direction of internal displacement of rock and soil mass, so it is unable to warn of the sliding direction of rock and soil mass, which seriously affects the accuracy and real-time of disaster warning and risk assessment. Finally, due to the scalability of the coaxial cable, the measurement range of the TDR technology is limited.

BOTDR technology was originally used to measure the surface displacement of geological mass. Now it has been gradually used to measure the underground displacement of rock and soil [17,18,19,20], due to the following features: (1) high stability, good electromagnetism immunity, corrosion resistance, high temperature resistance, etc.; (2) light weight, small size, suitable for a variety of geometric shapes. For example, the team of Bin Shi at Nanjing University applied fiber optic technology to deformation monitoring and strain measurement of rock and soil mass [21,22,23]. However, it still exists some deficiencies. Firstly, due to the strain conduction process between the fiber sensor and the measured object, the measurement accuracy of the sensor is seriously affected. Secondly, the measurement range of the BOTDR technology is small, and the fiber breaks when a large deformation occurs [24,25].

Borehole extensometers and settlement meters are often used for longitudinal displacement measurement of rock and soil mass. It is suitable for the initial stage of geotechnical deformation, which is characterized by small deformation and slow change rate [26,27,28]. In the sliding stage of landslide, the horizontal displacement of rock and soil mass is the main representation, so the borehole extensometer and settlement monitor are basically unsuitable for the later monitoring of the landslide.

In summary, at present, inclinometer, extensometer, subsidence meter and optical fiber sensor are mainly used to monitor the deformation of the underground rock and soil mass in a single direction at home and abroad. Although the above technologies have their own characteristics, they are difficult to achieve three-dimensional measurement of deep deformation of rock and soil mass, and there are many limitations such as low measurement accuracy, complex monitoring technology, inability to adapt to large deformation monitoring and expensive instruments. There are obvious limitations in practical use.

Our research team has carried out a series of basic research work on the principles, methods, models, devices, circuits and systems of deep deformation monitoring of landslides, in order to break through the limitations of the existing monitoring techniques [29,30,31]. It is worth stressing that in recent years a new method of deep displacement measurement based on integrated sensing of electromagnetic measurements has been proposed, and the corresponding two-dimensional and three-dimensional measurement sensors have been developed, which have the following features: (1) With the flexible array structure design of sensing unit, each sensing unit can slide freely with the surrounding rock and soil mass, and the deformation coordination is good. (2) Compared to the conventional deep displacement measuring instruments (such as inclinometer and TDR), our designed sensor has a larger measuring range, and higher measurement and positioning accuracy. (3) Simple structural design, easy to manufacture, durable and relatively low in cost. (4) Easy to realize remote, real-time and networked automatic monitoring. For these sensors, we have carried out in-depth research on sensor theory, measurement modeling, performance analysis and testing [32,33,34,35,36].

For our proposed deep displacement 3D measuring sensor, the measurement ranges of horizontal and vertical displacement between two adjacent sensing units (height of the sensing unit is 70 mm) is 0–30 mm, which can meet the monitoring requirements of most landslides, but not necessarily able to meet the monitoring requirements of some large-scale landslides in the fast-changing or near-sliding stage, thus limiting the sensor’s overall effectiveness on landslide early warning monitoring. At the same time, in order to adapt to the harsh and complex deep environment of rock and soil mass, the measuring device needs to have strong water-proof, anti-corrosion and compression resistance performance. However, in our previous design of sensing unit, in order to improve the displacement measurement range, the permanent magnet, Hall sensor and related PCB board are installed at the end face of sensing unit, which is not conducive to the implementation of water-proof, reinforcement and other protection means.

Therefore, in this paper, firstly the sensing unit structure of the corresponding 3D sensor is optimized by 3D printing and other technologies, which makes it more durable, less corrosive and more suitable for the actual needs of deep displacement measurement of rock and soil mass. Next, for the sensor with optimized structure, the relationship between the deep deformation 3D coordinates of rock-soil mass and the characteristic physical quantities (mutual inductance voltage, Hall voltage, tilt measuring voltage, etc.) has been further studied by combining theoretical analysis, numerical simulation and experimental testing. A new mathematical model for 3D measurement of deep displacement has been proposed, which is based on data analysis-contour query and polynomial least-squares curve fitting. By virtue of this measurement model, the output values of mutual inductance voltage, Hall voltage and tilt measuring voltage measured by the sensing units can be converted into the changes of relative horizontal displacement, vertical displacement and axial tilt angle between any two adjacent sensing units in real time. Finally, a simulation experiment platform has been established to simulate the three-dimensional movement characteristics of underground rock and soil, and a series of related experiments been conducted. Through comprehensive comparisons between the experimental testing and theoretical simulation results, the effectiveness of the structural optimization and the accuracy of the measurement model are preliminarily verified. Among them, the measurement error of inclination angle is less than 1°, the measurement errors of relative horizontal displacement and vertical displacement are less than 1.5 mm and the measurement range of horizontal displacement and vertical displacement has expanded to 50 mm at the same time. It shows that our proposing 3D sensor with updated structure design and measurement algorithm has higher measurement accuracy, larger measurement range, better waterproof, anti-corrosion and pressure resistance performance and can better meet better the needs of high-precision monitoring at the initial stage of rock and soil deformation and large deformation monitoring at the rapid change and imminent-sliding stage.

## 2. Sensor Structure and Working Principle

**Figure 1:**
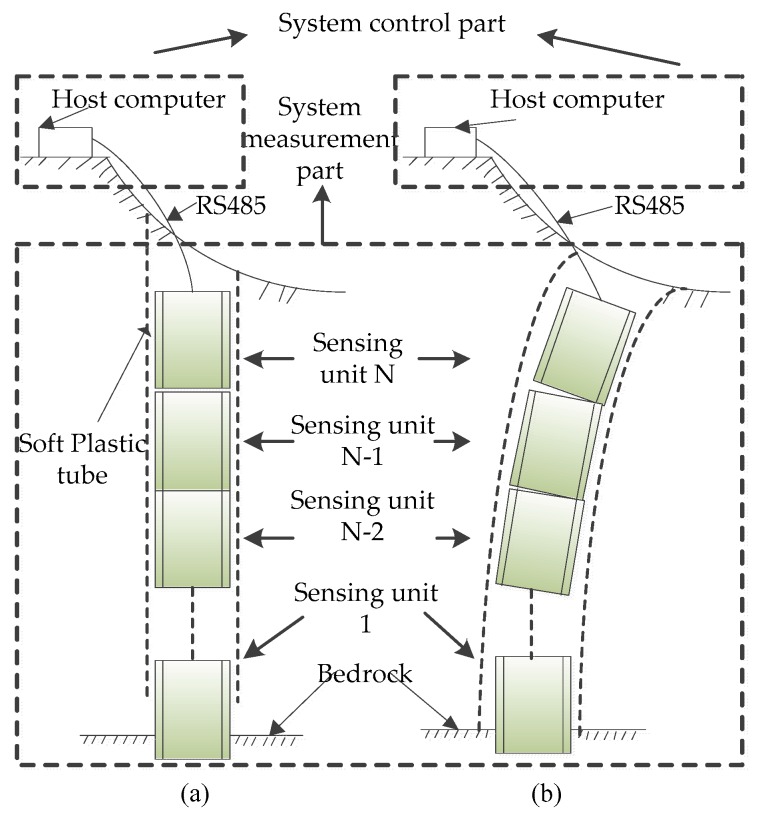
Overall structure of the system. (**a**) Non-sliding rock-soil mass; (**b**) sliding rock-soil mass

As shown in Figure 1, the magneto-electric deep displacement measurement sensor is composed of control part and measurement part. Data exchange between them is carried out by RS-485 bus. The control unit is the host computer, which transmits the voltage measurement command to the measurement part through RS-485 bus, and receives data such as mutual inductance voltage, Hall voltage and tilt measuring voltage fed back by the measurement part. The received data are input into a deep displacement measurement model to solve the relative tilt angle and relative displacement between any two adjacent units. The host computer can further transmit data to the remote client through wireless network. The proposed sensor is characterized by real-time, dynamic, remote and automatic.

The measuring part consists of N identical integrated sensing and measuring units (hereinafter referred to as sensing units). Each sensing unit has the same structure. In the practical engineering, the bottommost sensing unit is buried into the rock and soil fixed layer, numbered 1, serving as the reference point for the entire measurement system. From bottom to top, each sensing unit is numbered 1-N, respectively. When the system works, two adjacent sensing units form a measurement unit, such as: No.1 measurement unit is composed of sensing units 1 and 2, No.2 measurement unit composed of sensing units 2 and 3, …, with a total of N-1 measurement units for the sensor. When the instrument is installed on site, the upper and lower end faces of two adjacent sensing units can be directly attached or vertically spaced at a certain distance according to monitoring requirements. By measuring the displacement of each layer of rock and soil from N-1 measuring units in turn, the distributed flexible measurement of three-dimensional displacement from the surface to the depth of rock and soil mass can be realized.

**Figure 2:**
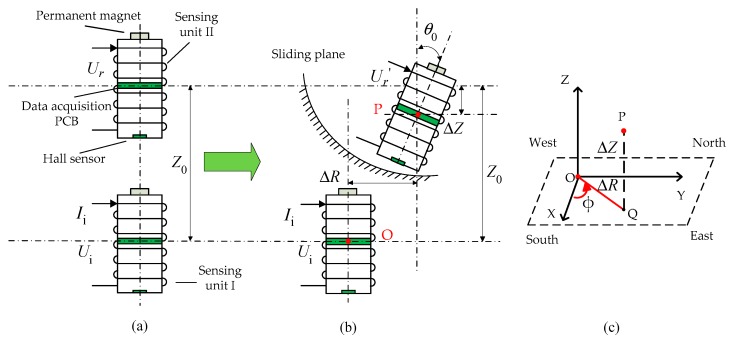
Schematic diagram of the previously designed measuring unit. (**a**) Initial geometrical setting; (**b**) occurrence of relative horizontal displacement Δ*R*, vertical displacement Δ*Z* and tilt angle *θ*_0_; (**c**) relative 3D position between points O and P.

Figure 2 shows the prototype of the magneto-electric deep displacement 3D measuring sensor, which we designed before [32,33,34,35,36]. Each sensing unit has the same structure. The main body is a solenoid, the outer wall is covered with several layers of thin coils and the inner wall is embedded by a PCB panel, which mainly integrates sinusoidal signal generation, mutual inductance voltage measurement, tilt measurement, serial communication and other functions. A small cylindrical permanent magnet with a diameter of 5.6 mm and a height of 18 mm is mounted at the center of the sensing unit’s lower end faces. Another PCB panel is mounted on the upper end faces of the sensing unit, and a micro Hall sensor is installed at the center of the panel, beside which a Hall voltage measuring integrated circuit is arranged. As mentioned, when the measurement system works, any two adjacent sensing units works as a measurement unit, for which, the lower sensing unit acts as a reference end while the upper one as a measurement end, and are labeled as Sensing units I and II, respectively. Here we only outline its working principle. More detailed description can refer our published works [32,33,34,35].

As shown in Figure 2, driven by the movement of surrounding rock and soil mass, a relative horizontal displacement Δ*R*, vertical displacement Δ*Z* and tilt angle *θ*_0_ might synchronously occur between Sensing Units I and II. The mutual inductance voltage *U_r_* and Hall voltage *U*_h_ generated on Sensing unit II will be varied simultaneously due to electromagnetic induction and Hall effect. We will explain it briefly.

If a sinusoidal voltage *U*_i_ with fixed amplitude and frequency, such as 10 KHz, is applied between the two ends of the solenoid in the sensing unit I, a corresponding mutual inductance voltage *U*_r_ will be generated across solenoid of sensing unit II, which, as we had proved [32], is directly proportional to the mutual inductance *M* between Solenoids I and II, with the following relationship

(1) $$U_{r}=\frac{U_{i}}{L}M$$

where, *L* is the coefficient of self-inductance. Mutual inductance *M*, reflecting the magnetic coupling state between Solenoids I and II, is determined by the solenoids’ geometry parameter (shape, size, turns of coils, etc.) and the relative position between them. Each solenoid is covered with thick polypropylene plastic pipe (featured as tough and resist to wear, corrosion, pressure, etc.), so generally it will not deform with the sliding of surrounding rock and soil mass, and the variation of *M* is only related to the relative displacement between two adjacent solenoids. In other words, the mutual inductance voltage *U*_r_ has a definitive functional relationship with the relative horizontal displacement Δ*R*, vertical displacement Δ*Z* and tilt angle *θ*_0_ between Sensing Units I and II. It can be generally expressed as

(2) $$U_{r}=f_{1}(\Delta R,\Delta Z,\theta_{0})$$

As Figure 2 shows, when Solenoids I and II produce a relative displacement (Δ*R*, Δ*Z* and *θ*_0_), the relative position changes between the permanent magnet on Sensing unit I and the Hall sensor on Sensing unit II, so the magnetic field strength and direction applied on the Hall sensor change accordingly, resulting in a varied output of Hall voltage from the Hall sensor. It can also expressed as

(3) $$U_{h}=f_{2}(\Delta R,\Delta Z,\theta_{0})$$

Meanwhile, by virtue of the built-in tilt measuring integrated module, the relative axial tilt angle *θ*_0_ between Sensing units I and II can be automatically measured with a measurement error of less than 1°. Therefore, as long as an effective measurement model can be established that is capable of accurately describing the complex relationship between the output values of *U*_r_, *U*_h_, *θ*_0_ from sensing units and the measuring displacement parameters Δ*R* and Δ*Z* between Sensing Units I and II, the relative horizontal displacement Δ*R* and vertical displacement Δ*Z* for each measuring unit can be inversely deduced (the axial tilt angle *θ*_0_ can be directly measured) according to the output values of mutual inductance and Hall voltage.

In our previous research work [35], we had established such a measurement model. It is an original deep horizontal and vertical displacement joint inversion method called “joint forward simulation-optimization inversion method”. It can realize a joint inversion of deep horizontal displacement and vertical displacement for the proposed sensor. The deviations between the experimentally measured and modeling inversed Δ*R* and Δ*Z* were tested to be less than 3 mm and 1 mm, respectively when the measuring range of Δ*R* and Δ*Z* controlled within 0–30 mm for each measuring unit.

Finally, it should be mentioned, the three-dimensional angular displacement can be expressed by Euler angles, that is, elevation angle *θ*_0_, yaw angle *ϕ* and roll angle *γ*. Considering the axisymmetric structure of sensing unit, the measurement of the roll angle *γ* is of little significance. In this context, the pitch angle *θ*_0_ and the yaw angle *ϕ* correspond to the relative axial tilt angle *θ*_0_ and the azimuth angle *ϕ* between two adjacent sensing units. The relative horizontal displacement Δ*R* and relative vertical displacement Δ*Z* can be calculated by combination of the two corresponding independent equations based on the measured mutual inductance voltage *U*_r_ and Hall voltage *U*_h_. At the same time, as shown in Figure 2c, the components on the *x*-axis and *y*-axis of ∆*R* can be calculated with ∆*R* and azimuth angle *ϕ*, as follows:

∆*X* = ∆*Rcosϕ* (4)

∆*Y* = ∆*Rsinϕ* (5)

Therefore, the correspondence between known parameters and unknown parameters is:

(∆*R*, ∆*Z*) = *f* (*U*_r_, *U*_h_, *θ*_0_) (6)

(∆*X*, ∆*Y*) = *f* (∆*R*, *ϕ*) (7)

As shown above, ∆*X* and ∆*Y* can be calculated by ∆*R* and *ϕ*, so unless otherwise specified, the deep displacement three-dimensional parameters to be measured are expressed by the relative horizontal displacement ∆*R*, relative vertical displacement ∆*Z*, relative tilt angle (*θ*_0_) and relative direction angle (*ϕ*) between adjacent sensing units. The relative tilt angle *θ*_0_ and relative direction angle *ϕ* can be directly measured by PCB integrated module, therefore the deep displacement measurement model is simplified as applying two independent functions (Equations (2) and (3)) to solve two independent variables (∆*R*, ∆*Z*). In the practical monitoring process, if the surrounding rock and soil slide, the above-mentioned *U*_r_, *U*_h_, *θ*_0_ and *ϕ* can be measured in real time by the proposed 3D sensor. From them, not only the relative vertical displacement and horizontal displacement can be calculated, but also the relative tilt angle and tilt direction of the two sensing units can be determined, so as to realize the three-dimensional measurement of deep displacement of rock and soil mass.

## 3. Optimization of Sensor Structure and Establishment of Mathematical Mode

### 3.1. Sensing Unit Structure Optimization Design

Combined theoretical research and experimental results of our research group, shortcomings of the above structure design are found. First of all, our previous research results show that, based on the above structural design of the deep displacement measurement senor, the measured relative tilt angle of any measurement unit can reach 0–90°, but the measuring ranges of relative horizontal displacement and vertical displacement can only reach 0–30 mm. It will limit the effectiveness of the proposed system for landslide early warning and monitoring to a certain extent, considering the big measurement range requirements of some large landslides disasters in near and accelerated sliding stage. Secondly, the PCB board and the permanent magnet have been mounted on the upper and lower end faces of the sensing unit respectively. Originally, its purpose is to narrow the initial distance between the permanent magnet and Hall sensor in each measurement unit and increase the displacement measurement range. However, it may affect the durability of the designed sensing unit due to not conducive to water-proof and pressure-proof treatments and increase difficulty in the subsequent sealing packaging and overall assembly of sensors for in-situ monitoring.

**Figure 3:**
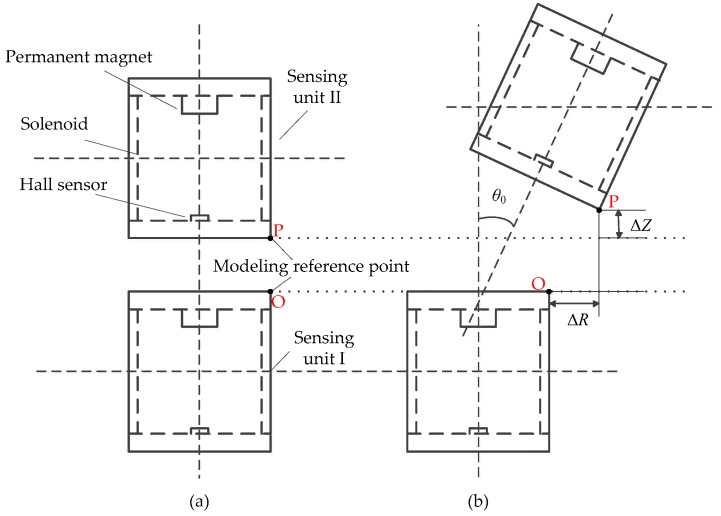
Schematic diagram of the optimized measurement unit. (**a**) Initial geometrical setting; (**b**) occurrence of relative horizontal displacement Δ*R*, vertical displacement Δ*Z* and tilt angle *θ*_0_.

**Figure 4:**
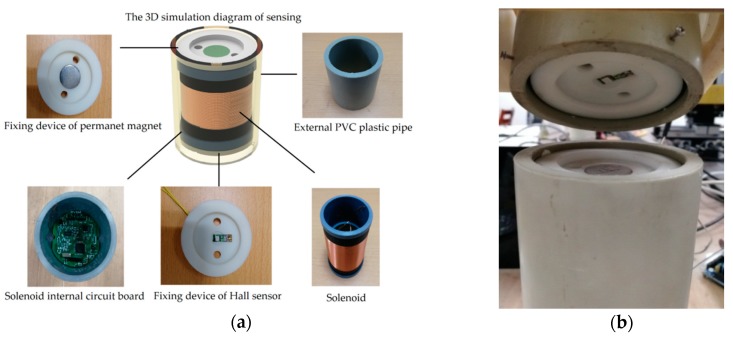
Updating structure design of sensing unit based on 3D printing. (**a**) 3D simulation model of sensing unit structure based on 3D CAD/CAM software and physical figure of each component; (**b**) photograph of device structure.

In order to adapt to the complex environment of deep rock and soil mass, the sensing unit must have such properties as waterproof and anti-corrosive. The structural optimization design of sensing unit for the sensor is required. Firstly, as shown in Figure 3, the permanent magnet and the Hall sensor are no longer installed on the upper and lower end faces of sensing unit, but are moved a certain distance inside, respectively. In this way, the permanent magnet and Hall sensor do not protrude from the upper and lower end faces of the solenoid. At the same time, since the external PVC plastic pipe is 20 mm higher than the solenoid, the glue can be filled in the gap between the end face of the solenoid and the end face of the external PVC plastic pipe, so that the sensing unit has better waterproof and anti-corrosive properties. Figure 4 shows the actual structure of each component of the sensing unit in details.

**Figure 5:**
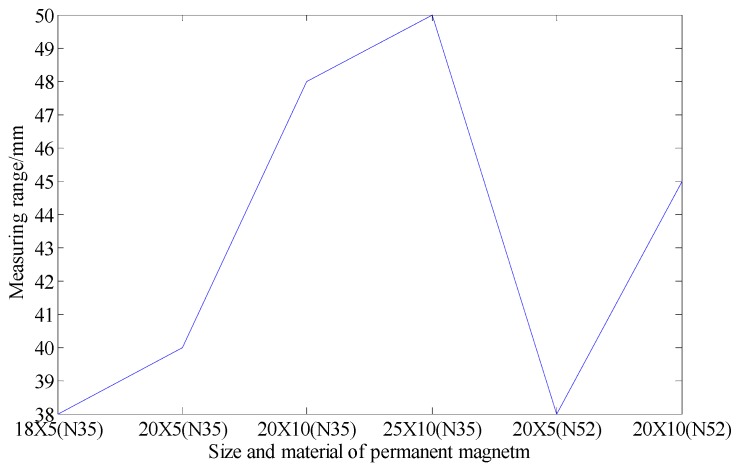
Comparison of deep displacement measurement range and permanent magnet size. “20X5”means the size of permanent magnet is 20 mm in diameter and 5 mm in height, and so on. “N35” and “N52” represent different materials of the permanent magnet.

At the same time, in order to improve stability of the device and ensure the initial positions of three center points of the permanent magnet, Hall sensor induction surface and solenoid surface to be in the same vertical line, we use 3D printing technology to design the fine structure for the placement and fixing of permanent magnets, Hall sensors and PCB boards. The fixing devices of permanent magnet and Hall sensor can not only enhance the robustness and durability of the sensing unit on the one hand, but also prevent the permanent magnet or the Hall sensor from falling or shifting, which will increase the measurement error and even lead the measurement system failing to work. Compared to the original design, the renewed device is robust, stable and convenient for assembly. Secondly, the structure of permanent magnets is optimized. Our research shows that the deep displacement of rock and soil mass can be accurately measured by using mutual inductance effect and Hall effect synthetically. However, the measurement range based on Hall effect is smaller than that based on mutual inductance effect, that is, the system’s measurement range of the system is mainly limited by the Hall effect. Under certain conditions, the increase of magnetic field intensity of the permanent magnet can increase the measuring range of Hall voltage. Based on the geometry of mutual inductance solenoids, a large number of experiments have been carried out on permanent magnets with different sizes and magnetic field intensities, and the comparison results are shown in Figure 5. It can be seen in Figure 5 that with the same magnetic brand, as the size of the permanent magnet becomes larger, the actual measurement range of the Hall sensor increases accordingly. Moreover, with the same size of permanent magnet, the measurement range of using the N35 permanent magnet is larger than using the N52 permanent magnetic (for example, 20X5 (N35) is greater than 20X5 (N52)). The conclusion also verified the structural principle of the related sensing model (referred as Hall voltage measuring modeling), which has been detailed in our published paper [34]. The Hall sensor saturated all the time when the permanent magnet has too strong magnetism because of the measurement range of Hall sensor (SS94A1F, made by Honeywell International, Morris Plains, NJ,, USA.) is −100 Gauss to 100 Gauss. Therefore, the size of the permanent magnet corresponding to the largest location in the figure is selected, as shown in Table 1. However, it should be pointed out that although the structure of the sensing unit has been optimized, the measurement principle remains unchanged.

### 3.2. Mathematical Modeling

#### 3.2.1. Experimental Data Acquisition

**Figure 6:**
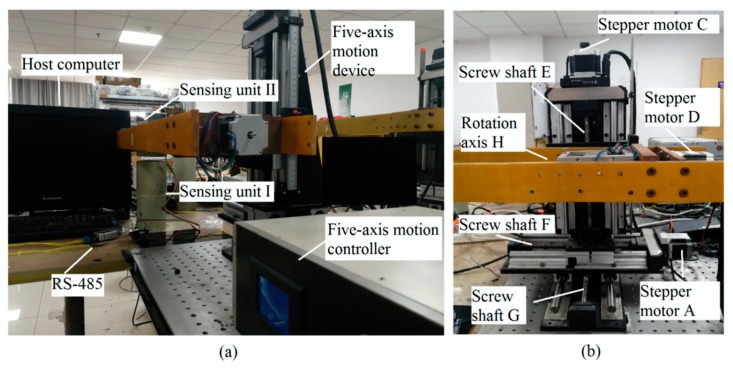
Data acquisition experimental device. (**a**) The system composition of experimental device; (**b**) five-axis motion device.

**Figure 7:**
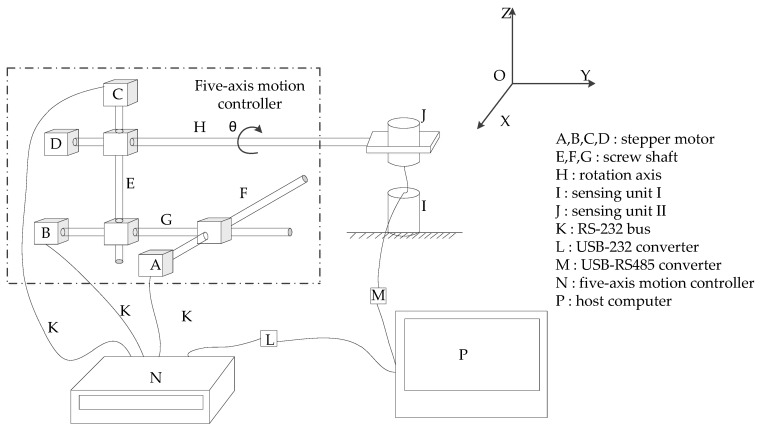
The schematic diagram of data acquisition experimental device.

The mutual inductance voltage and Hall voltage data are collected as benchmark data for establishing the new mathematical model when calculating the displacements. A photograph of data acquisition experimental platform is shown in Figure 6. It consists of host computer, five-axis motion device, controller of motion device and sensor prototype (mainly includes a measuring unit and its communication setting with the host computer). The host computer communicates with the sensor prototype through RS-485 and communicates with the controller through RS-232. Figure 7 shows schematic diagram of the above experimental device. Sensing unit I is fixed on the smooth surface of the test bench. Three stepper motors (*A*, *B* and *C*) drive sensing unit II to do translational motion in *X, Y* and *Z* axes respectively, which can simulate three-dimensional space motion. At the same time, controlled by motor *D,* the rotation-axis *H* can drive the sensing unit II to rotate axially (*θ*_0_), simulating the case of inclination. The motion controller can receive the command from the host computer to control the motion of five-axis motion device. However, it should be pointed out, for convenience of experiment and data analysis, the relative direction angle *ϕ* has been set as 0 in this experiment process, that is, ∆*X* = ∆*R* and ∆*Y* = 0 according to Equations (4) and (5). Therefore, the horizontal displacement ∆*R*, vertical displacement ∆*Z* and tilt angle *θ*_0_ between adjacent sensing units can be changed by driving motor *A, C* and *D,* respectively, so as to simulate the deep displacement of rock and soil mass.

For any measurement unit, the reference points taken by the experiment are the bottom edge points of the upper sensing unit and the upper edge points of the lower sensing unit, namely, points P and O, as shown in Figure 3. The data acquisition process of mutual inductance voltage and Hall voltage is as follows. Firstly, the host computer sends instructions to the reference end of measuring unit (Sensing unit I in Figure 2 and Figure 3) to generate sinusoidal waves which will input into the solenoid of Sensing unit I. Secondly, the host computer sends instructions to the measuring end of measuring unit (Sensing unit II in Figure 2 and Figure 3) to measure the mutual inductance voltage *U*_r_ which generated in the solenoid of sensing unit II. Thirdly, the host computer sends instructions to the reference end of measuring unit to turn off the sinusoidal wave so as to reduce the interference of Hall measurement. Finally, the host computer sends instructions to the measurement end to measure Hall voltage *U*_h_. When one measurement of *U*_r_ and *U*_h_ is completed, an instruction to change the horizontal or vertical displacement is sent to the five-axis motion controller. The displacement change is 1 mm each time. The time interval T = 3 s for each data sampling point (one data acquisition of mutual inductance voltage and Hall voltage), and the experimental process and data storage are controlled by the host computer.

**Figure 8:**
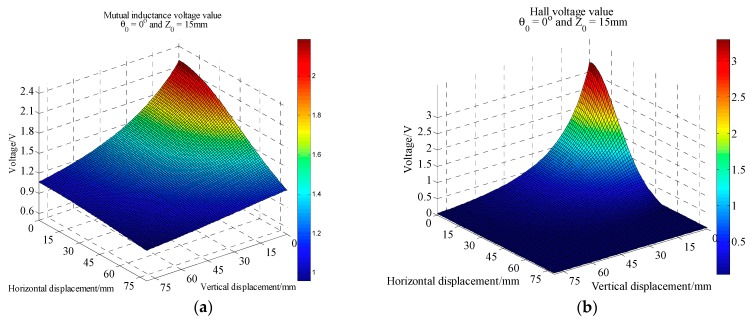
Voltage acquisition data at 0° inclination. (**a**) Mutual inductance voltage; (**b**) Hall voltage.

**Figure 9:**
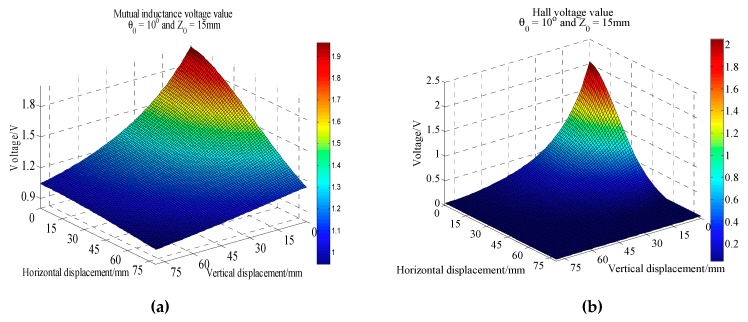
Voltage acquisition data at 10° inclination. (**a**) Mutual inductance voltage; (**b**) Hall voltage.

**Figure 10:**
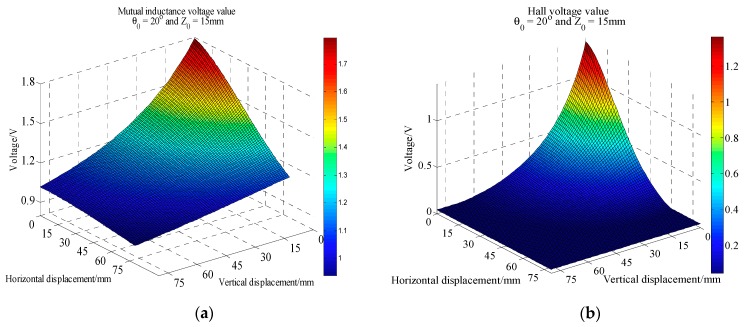
Voltage acquisition data at 20° inclination. (**a**) Mutual inductance voltage; (**b**) Hall voltage.

According to the different tilt angle *θ*_0_, many batches of mutual inductance and Hall voltage data acquisition experiments were carried out. In each batch of data acquisition process, the five-axis motive device moves upward 80 mm vertically and outward 80 mm horizontally. Each data acquisition experiment takes about 8 h. The initial vertical distance between these two sensing units can be set flexibly according to experimental requirements. Here it is 15 mm. The whole experiment is controlled by host computer. Figure 8, Figure 9 and Figure 10 are 3D curves of mutual inductance voltage and Hall voltage data measured at tilt angles of 0, 10 and 20 degrees, respectively. Figure 8a, Figure 9a and Figure 10a show the mutual inductance voltage data. Figure 8b, Figure 9b and Figure 10b show the Hall voltage data.

**Figure 11:**
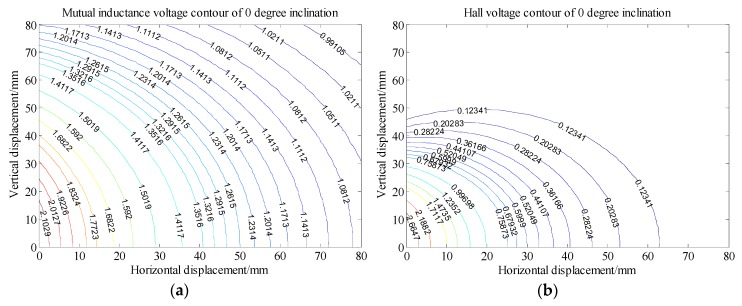
Voltage contour at 0° inclination. (**a**) Mutual inductance voltage contour; (**b**) Hall voltage contour.

**Figure 12:**
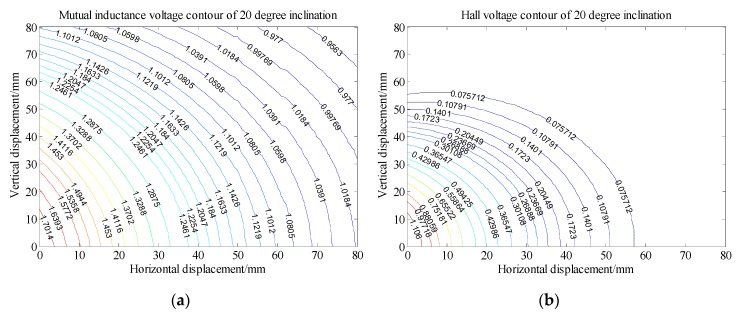
Voltage contour at 20° inclination. (**a**) Mutual inductance voltage contour; (**b**) Hall voltage contour.

Analysis of Figure 8 shows that, under 0 degree of tilt angle (*θ*_0_ = 0°)*,* the mutual inductance voltage and Hall voltage measured at a certain time correspond to many (*x*, *y*, *U*) points in the graph, where *x* is horizontal displacement, *y* is vertical displacement and *U* is the corresponding voltage value. These points can be connected by a smooth curve to form a voltage contour. Thus, all the mutual inductance voltage and Hall voltage data measured at 0 degrees inclination correspond to a set of contours, among them each voltage data corresponds to one voltage contour, and the *x*-axis of the contour corresponds to the horizontal displacement, the *y*-axis corresponds to the vertical displacement, as shown in Figure 11. The contour shows that the single parameter of mutual inductance voltage or Hall voltage can hardly achieve effective deep displacement measurement of rock and soil mass. When Sensing unit II moves a specific displacement relative to Sensing unit I (e.g., Δ*R* = 10 mm and Δ*Z* = 10 mm), a corresponding mutual inductance voltage contour and Hall voltage contour can be obtained in Figure 11a,b, respectively. These two contours must have an intersection point (10, 10), which is the spatial position of Sensing unit II relative to Sensing unit I. Its horizontal and vertical coordinates correspond to the relative horizontal and vertical displacements between these two sensing units. Figure 12a,b are the contour maps of mutual inductance voltage and Hall voltage calculated respectively from the mutual inductance voltage and Hall voltage data in Figure 10a,b at the inclined angle of 20 degrees. These contours intersect in pairs and can also be used to determine the relative horizontal and vertical displacements between Sensing units I and II. Inspired by this, we attempt to establish the deep displacement measurement model of rock and soil mass by voltage contour modeling.

#### 3.2.2. Data Modeling

In the previous section, it is concluded that the intersection points of mutual inductance voltage contour and Hall voltage contour correspond to the relative displacements of adjacent sensing units. Therefore, the authors establish a specific voltage contour model to measure the deep displacement of rock and soil mass. In order to establish the contour model of mutual inductance voltage and Hall voltage, it is necessary to extract the discrete points of the voltage contour first, and then perform curve fitting on these extracted discrete points to obtain the contour function. Finally, the intersection points of the mutual inductance voltage curve and Hall voltage curve are solved to obtain the relative displacement of the adjacent sensing units.

The calibrated reference voltage data and the corresponding tilt angle *θ*_0_ are stored in two-dimensional array. Each tilt angle *θ*_0_ corresponds to two 80 * 80 two-dimensional arrays of *θ*-*X*-*Y*-*U*_r_ and *θ*-*X*-*Y*-*U*_h_. The row of the array represents the horizontal displacement is *n*_i_*1(where *n*_i_ is the label of the array rows, numbered 0–n, and the voltage data are collected once every 1 mm in the experiment), and the column of the array represents the vertical displacement is *m*_i_ * 1. Now we will take the mutual inductance voltage contour modeling as an example to introduce the displacement solution process.

Firstly, according to the mutual inductance voltage *U*_r_ and tilt angle *θ*_0_ measured by the microcomputer and uploaded to the host computer, the corresponding array *θ-X*-Y-*U*_r_ is searched, the first row of the array (*x*_r1_ = 0 × 1 = 0) is traversed and the value of the column corresponding to *U*_r_ is found (*y*_r1_ = *m*_i_ × 1), thus getting the first contour discrete point. Then, traverse each row of the array to get a set of discrete points of the contour:

$$\left\{(x_{r1},y_{r1}),(x_{r2},y_{r2}),(x_{r3},y_{r3}),(x_{r4},y_{r4}),\ldots ,(x_{\mathrm{rn}},y_{\mathrm{rn}})\right\}$$

When traversing a row of array, if the corresponding voltage *U*_r_ is intermediate between the two adjacent voltage values in the array, that is, *U*_r1_ ≥ *U*_r_ ≥ *U*_r2_, where *U*_r1_ corresponds to the coordinate (*x*_r1_, *y*_r1_), *U*_r2_ to (*x*_r1_, *y*_r2_) and *y*_r2_ − *y*_r1_ = 1 (the displacement step value is 1 mm), then it is assumed that in the interval [*y*_r1_, *y*_r2_], there is a linear relationship between the vertical displacement and the voltage, i.e., *y*_r_ = *y*_r1_ + (*U*_r1_ − *U_r_*)/(*U*_r1_ − *U*_r2_). By substituting (*U*_r1_, *y*_r1_), (*U*_r2_, *y*_r2_) and *U*r into this expression, the values of *y*_r_ can be solved, and the discrete point (*x*_r_, *y*_r_) can be obtained.

According to the mutual inductance voltage, Hall voltage and tilt angle data uploaded by the microcomputer, find the corresponding arrays of *θ*_0_-*X-Y-U*_r_ and *θ*_0_-*X-Y-U*_h_, traverse the arrays, and get the discrete points of the mutual inductance voltage contour and Hall voltage contour. To obtain the relative displacement of adjacent sensing units, it is required to perform curve fitting on the obtained discrete points to obtain a voltage contour function. The authors use a polynomial least squares method to fit the curve function.

The fitting function is:

(8) $$\omega (x)=a_{0}\omega_{0}(x)+a_{1}\omega_{1}(x)+\cdots +a_{n}\omega_{n}(x)=\sum_{j=0}^{n}a_{j}\omega_{j}(x)$$

Need to meet the fitting conditions:

(9) $$\sum_{i=1}^{m}{\left[\omega (x_{i})-y_{i}\right]}^{2}=\sum_{i=1}^{m}{\left[\sum_{j=0}^{n}a_{j}\omega_{j}(x_{i})-y_{i}\right]}^{2}$$

The function class $\left\{\omega_{0}(x),\omega_{1}(x),\omega_{2}(x),\cdots ,\omega_{n}(x)\right\}$ is set to $\left\{1,x,x^{2},\cdots ,x^{n}\right\}$.

Then there is

(10) $$\omega (x)=a_{0}+a_{1}x+a_{2}x^{2}+\cdots +a_{n}x^{n}\left(n+1<m\right)$$

The following equations are voltage contour curve equations obtained by fitting the obtained discrete points when the horizontal displacement and the vertical displacement are both 10 mm. The fitting function is a 6th order polynomial. Equations (11) and (12) are the contour curve fitting equations of mutual inductance voltage and Hall voltage, respectively.

(11) $$y_{r}=0.000000014x^{6}-0.0000016x^{5}+0.000068x^{4}-0.0013x^{3}+0.033x^{2}-0.00398x+10.8$$

(12) $$y_{h}=-0.00000026x^{6}+0.000014x^{5}-0.000286x^{4}+0.00248x^{3}-0.0264x^{2}+0.015x+11.73$$

**Figure 13:**
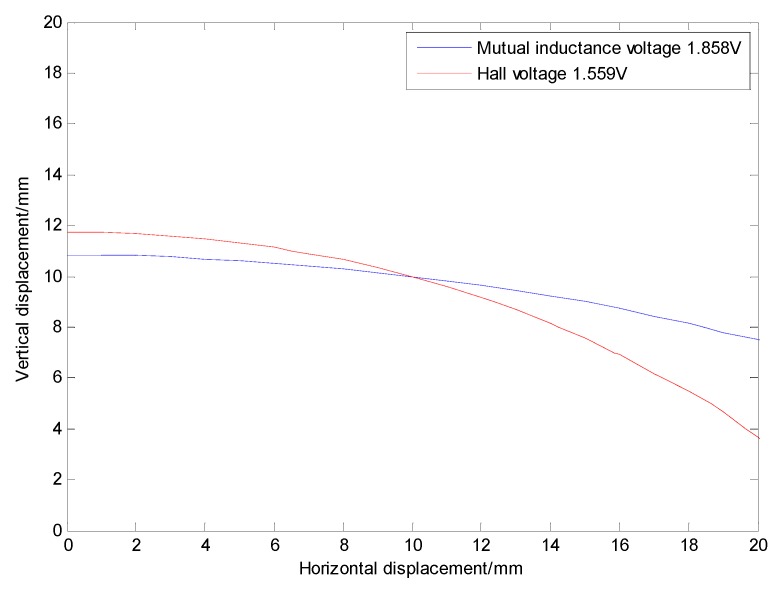
Intersection of two contours when the horizontal displacement and the vertical displacement are both 10 mm.

**Figure 14:**
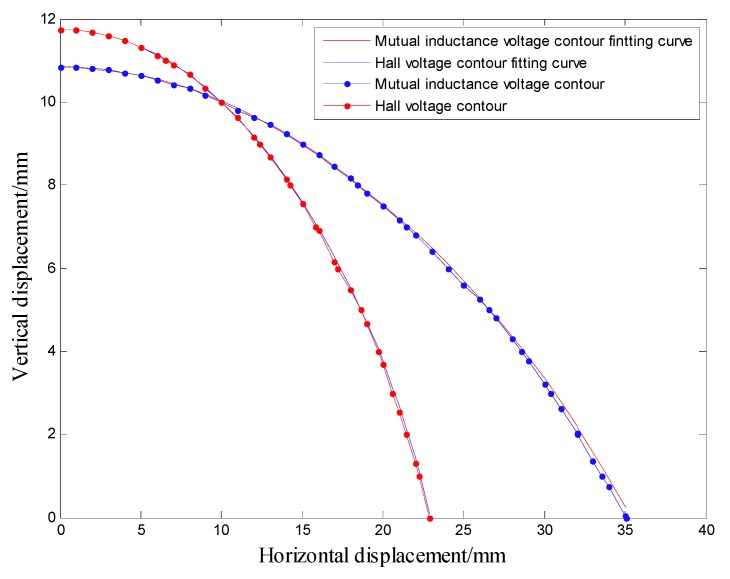
Contour comparison before and after fitting.

Figure 13 shows the voltage contour drawn by MATLAB when the measured mutual inductance voltage is 1.858 V, Hall voltage is 1.559 V and the horizontal displacement and vertical displacement are both 10 mm under 0 degree of tilt angle. The fitted contour curve and the original contour discrete points of the mutual inductance voltage and Hall voltage are shown in Figure 14. Solving these two equations, the solutions are:

*x* = 9.916 mm, *y* = 10.036 mm

## 4. Experiment and Analysis

First of all, we will conduct experiments to simulate the three-dimensional displacement of Sensing unit II relative to Sensing unit I in the deep rock and soil mass. Applying the new measurement model, the measured values of mutual inductance voltage, Hall voltage and tilt measuring voltage of between two adjacent sensing units can be solved as the relative horizontal displacement, vertical displacement and tilt angle in real-time. In the verification experiment, Sensing unit II is moved to be displaced relative to Sensing unit I through the motion controller, the displacement is recorded, and the mutual inductance voltage and Hall voltage at these corresponding points are measured. Experiments are carried out under different inclination angles and displacements. Among them, the change range of tilt angle is 0°–20°, that of horizontal displacement is 0–80 mm and that of vertical displacement is 0–80 mm.

We set up the 3D displacement measurement platform as shown in Figure 6 and Figure 7 to simulate the deformation of the measured object, and collect the mutual inductance voltage data and Hall voltage data as the benchmark data to establish the mathematical model when calculating the displacement. The experimental platform is composed of host computer, five-axis motion device with its controller and sensor prototype (measuring unit).

**Figure 15:**
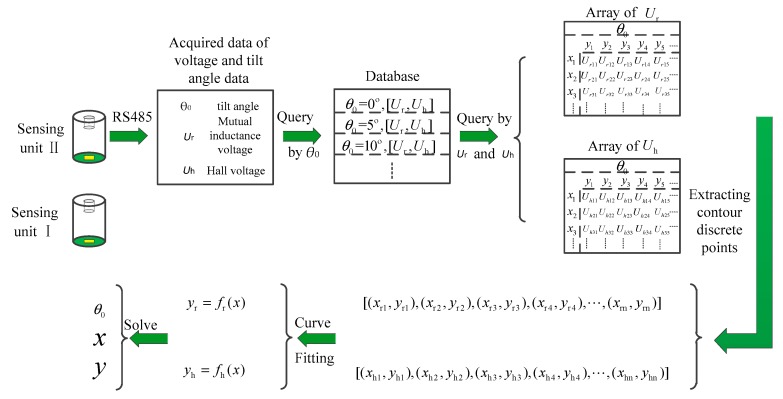
Flow chart for displacement solution.

Figure 15 shows a block diagram for solving the displacement based on the measured voltage. In the diagram, the discrete points of mutual inductance voltage contour and Hall voltage contour are obtained by inquiring database arrays according to the mutual inductance voltage, Hall voltage and tilt angle measured by Sensing unit II. Then these discrete points are fitted by polynomial least square method. Finally, the intersection points of the two fitting curves are obtained, and the solutions to *x* and *f*(*x*) are the horizontal and vertical displacements of these two adjacent sensing units.

To fully evaluate the validity and accuracy of the new measurement model, we compare the experimental testing results with the theoretical modeling calculation results under different tilt angles (*θ*_0_). Under the same tilt angle, the variation ranges of horizontal displacement and vertical displacement both are 0–50 mm, and the variation interval is 1 mm. Table 2, Table 3 and Table 4 show the comparison results under the conditions of 0°, 10° and 20° of *θ*_0_. Considering limitation of article length, to each table only 18 groups of combined data of [*x*, *y*] are randomly selected for comparative analysis. In each table, *x* and *y* represents the horizontal displacement and vertical displacement between Sensing units I and II, respectively. The first column of [*x*, *y*] are the experimental testing values, the second column are the theoretical calculated values, and the third column are the absolute errors between them. We made statistical analysis on the data in Table 2, Table 3 and Table 4. It indicates that when *θ*_0_ = 0°, the relative and absolute mean errors of horizontal displacement (*x*) are −0.352 mm and 0.605 mm, and the relative and absolute mean errors of vertical displacement (*y*) are 0.210 mm and 0.388 mm, respectively. When *θ*_0_ = 10°, they are −0.236 mm and 0.528 mm for x, 0.158 mm and 0.482 mm for *y*, respectively. When *θ*_0_ = 20°, they are 0.197 mm and0.582 mm for *x*, and 0.033 mm and 0.394 mm for *y*, respectively. Meanwhile, under each table, the absolute errors of horizontal displacement and vertical displacement are all less than 1.5 mm. Combined with more detailed verification experiment results carried out by specifying more tilt angles, while the measured *x* and *y* continuously changed, some common conclusions can be drawn: (1) Variations of tilt angle has little effect on the measuring errors of horizontal and vertical displacements (*x*, *y*); (2) when the measurement ranges of horizontal and vertical displacement are 0–50 mm, the absolute measurement errors of them both are less than 1.5 mm; (3) the measurement accuracy ofvertical displacement is slightly higher than that of horizontal displacement.

## 5. Conclusions

This paper mainly expands the effective measurement range of the three-dimensional displacement measurement sensor designed in the previous research, optimizes the sensor structure, redesigns the data acquisition experiment and rebuilds the mathematical model in view of the complex environment inside the rock and soil. Based on the theoretical analysis and experimental results, the following conclusions are obtained.

As described above, we optimized the structure of the sensing unit through 3D printing and other technologies, and improved the shape and material parameters of the permanent magnet through a large number of experiments. The results show that the improved sensor has better waterproof, anticorrosive and compressive-resistance properties, and is more suitable for the actual needs of deep displacement measurement of rock and soil mass. More importantly, the combination of structural optimization and updated measurement model broadens the measurement range of the sensing unit from 0–30 mm to 0–50 mm.

According to the different tilt angle, many batches of mutual inductance and Hall voltage data acquisition experiments have been conducted. Through in-depth analysis of the experimental data, based on the data query algorithm and the polynomial least square curve fitting theory, a new mathematical model for 3D measurement of deep displacement has been proposed. By virtue of it, the output values of mutual inductance voltage, Hall voltage and tilt measuring voltage measured by the sensing units can be converted into the changes of relative horizontal displacement, vertical displacement and axial tilt angle between any two adjacent sensing units in real time. Analyzing the results, the measuring errors of horizontal displacement and vertical displacement are tested to be 0–1.5 mm. In our published paper [35], the measurement model realized the joint inversion of deep horizontal displacement and vertical displacement for the proposed 3D sensor with measuring errors of 0–3 mm. Compared with the previous model, the measurement error of this new model has reduced by half.

It shows that our revised deep displacement 3D measuring sensor, based on the specific flexible array structure design of sensing units, after structural optimization and measurement algorithm improvement, has such advantages as higher measurement accuracy, larger measurement range, better waterproof, anti-corrosion and pressure resistance performance. It can better meet the needs of high-precision monitoring at the initial stage of rock and soil deformation and large deformation monitoring at the rapid change and imminent-sliding stage. It is more competitive in deep displacement 3D monitoring and geo-hazard prediction and early warming than most of the existing deep displacement monitoring instruments, such as inclinometers, settlement gauges, extensometers and optical fiber sensors.

In order to be more consistent with the actual landslide state and more accurate in early warning and prediction of landslide accidents, the measurement range of the device should still be considered in the future research. The research group is considering using different materials and different shapes of permanent magnets to increase the measurement range of the system. At the same time, we consider to reduce the measurement error and improve the system accuracy by optimizing the modeling scheme and making interference analysis, such as temperature, conductivity, geotechnical geology, water content and magnetoelectric interface.

## Figures and Tables

**Table 1:** Size and parameters for the selected permanent magnet.

Parameter	Value
Diameter	25 ± 0.1 mm
Thickness	10 ± 0.1 mm
Material	NdFeB
Grade Unit	N35
Remanent	12200 Gauss
Maximum Magenetic Energy Product	35 MGOe
Surface Magnetic Size	3450 Gauss

**Table 2:** Measuring result of [horizontal displacement, vertical displacement] of 0° tilt angle (unit: mm).

Actual Displacement	Calculated	Error
[10.000, 2.000]	[10.152, 1.941]	[0.152, −0.059]
[10.000, 5.000]	[9.897, 5.007]	[−0.103, 0.007]
[10.000, 10.000]	[9.916, 10.036]	[−0.084, 0.036]
[10.000, 15.000]	[10.001, 14.999]	[0.001, −0.001]
[10.000, 20.000]	[10.182, 19.941]	[0.182, −0.059]
[20.000, 5.000]	[19.969, 4.960]	[−0.031, −0.040]
[20.000, 15.000]	[19.841, 15.059]	[−0.159, 0.059]
[20.000, 25.000]	[19.027, 25.340]	[−0.973, 0.340]
[20.000, 35.000]	[18.776, 35.379]	[−1.224, 0.379]
[20.000, 45.000]	[19.291, 46.205]	[-0.709, 1.205]
[30.000, 10.000]	[29.964, 9.990]	[−0.036, −0.010]
[30.000, 20.000]	[30.667, 19.649]	[0.667, −0.351]
[30.000, 40.000]	[29.429, 39.285]	[−0.571, −0.715]
[30.000, 50.000]	[31.276, 49.638]	[1.276, −0.362]
[40.000, 20.000]	[39.198, 20.490]	[−0.802, 0.490]
[40.000, 40.000]	[38.922, 40.539]	[−1.078, 0.539]
[50.000, 20.000]	[48.649, 21.095]	[−1.351, 1.095]
[50.000, 40.000]	[48.507, 41.231]	[−1.493, 1.231]

**Table 3:** Measuring result of [horizontal displacement, vertical displacement] of 10° tilt angle (unit: mm).

Actual Displacement	Calculated	Error
[10.000, 2.000]	[9.963, 0.577]	[−0.037, −1.423]
[10.000, 5.000]	[9.876, 5.059]	[−0.124, 0.059]
[10.000, 10.000]	[9.679, 10.110]	[−0.321,0.110]
[10.000, 15.000]	[10.060, 14.992]	[0.060, −0.008]
[10.000, 20.000]	[10.132, 20.005]	[0.132, 0.005]
[20.000, 5.000]	[19.984, 5.002]	[−0.016, 0.002]
[20.000, 15.000]	[20.104, 14.925]	[0.104, −0.075]
[20.000, 25.000]	[19.442, 25.221]	[−0.558, 0.221]
[20.000, 35.000]	[19.500, 35.117]	[−0.500, 0.117]
[20.000, 45.000]	[20.555, 44.922]	[0.555, −0.078]
[30.000, 10.000]	[30.019, 9.986]	[0.019, −0.014]
[30.000, 20.000]	[30.385, 19.774]	[0.385, −0.226]
[30.000, 40.000]	[28.583, 41.016]	[−1.417, 1.016]
[30.000, 45.000]	[28.795, 46.329]	[−1.205, 1.329]
[40.000, 20.000]	[39.988, 20.053]	[−0.012, 0.053]
[40.000, 40.000]	[41.378, 38.905]	[1.378, −1.095]
[50.000, 20.000]	[48.541, 21.431]	[−1.459, 1.431]
[50.000, 40.000]	[48.774, 41.412]	[−1.226, 1.412]

**Table 4:** Measuring result of [horizontal displacement, vertical displacement] of 20° tilt angle (unit: mm).

Actual Displacement	Calculated	Error
[10.000, 2.000]	[10.374, 1.849]	[0.374, −0.151]
[10.000, 5.000]	[10.425, 4.828]	[0.425, −0.172]
[10.000, 10.000]	[10.093, 9.956]	[0.093, −0.044]
[10.000, 15.000]	[10.279, 14.900]	[0.279, −0.100]
[10.000, 20.000]	[11.105, 19.700]	[1.105, −0.300]
[20.000, 5.000]	[20.204, 4.867]	[0.204, −0.133]
[20.000, 15.000]	[19.984, 14.988]	[−0.016, −0.012]
[20.000, 25.000]	[19.221, 25.372]	[−0.779, 0.372]
[20.000, 35.000]	[19.912, 35.144]	[−0.088, 0.144]
[20.000, 45.000]	[21.074, 45.742]	[1.074, 0.742]
[30.000, 10.000]	[30.428, 10.190]	[0.428, 0.190]
[30.000, 20.000]	[30.171, 19.854]	[0.171, −0.146]
[30.000, 40.000]	[29.114, 40.506]	[−0.886, 0.506]
[30.000, 50.000]	[28.755, 51.328]	[−1.245, 1.328]
[40.000, 20.000]	[40.294, 19.719]	[0.294, −0.281]
[40.000, 40.000]	[41.226, 39.158]	[1.226, −0.842]
[50.000, 20.000]	[49.548, 20.559]	[−0.452, 0.559]
[50.000, 40.000]	[51.331, 38.926]	[1.331, −1.074]
